# Successful Leadless Pacemaker Implantation in an Elderly Patient With Dextrocardia and Situs Inversus

**DOI:** 10.7759/cureus.17858

**Published:** 2021-09-09

**Authors:** Yoshiyasu Aizawa, Tomoko Ichiki, Akihiro Yoshizawa, Yuto Monma, Takashi Nakayama, Tomoko S Kato, Shigenobu Inami, Yoshihide Fujimoto, Akio Kawamura

**Affiliations:** 1 Cardiology, International University of Health and Welfare Narita Hospital, Narita, JPN

**Keywords:** atrio-ventricular block, leadless pacemaker, micra, dextrocardia, situs inversus

## Abstract

Leadless pacemaker is indicated in patients with symptomatic bradycardia as an alternative therapy when transvenous pacemaker implantation is considered difficult or at high risk. The experience of implanting leadless pacemaker in patients with dextrocardia and situs inversus is limited. A 94-year-old male was transferred to our hospital due to advanced atrio-ventricular block with episode of syncope. Chest radiograph and computed tomography revealed dextrocardia with situs inversus. Emergency cardiac catheterization was performed and a temporary pacemaker was inserted, but the patient removed it due to delirium. So, a leadless pacemaker was implanted to him. Shorter time of bed-rest after the implantation and shorter hospital stay would be beneficial of implanting a leadless pacemaker. Precise anatomical evaluation would be important to perform implantation efficiently and safely.

## Introduction

Permanent cardiac pacing is a gold standard therapy for symptomatic bradycardia. A leadless intracardiac transcatheter pacing system is also available with advantages of fewer lead problems and less chance of device infections. Dextrocardia with situs inversus is a rare condition in which the heart and major visceral organs are positioned on the right side of the body. The incidence of dextrocardia with situs inversus is estimated to be 0.0125% in the general population [1]. Although cardiac complications sometimes exist, most affected individuals can live a normal life. Our case was complicated with advanced atrio-ventricular block, which does not seem to be a complication of dextrocardia. Due to its extremely rare incidence, the experience of leadless pacemaker implantations in patients with dextrocardia and situs inversus is very limited.

## Case presentation

**Figure 1:**
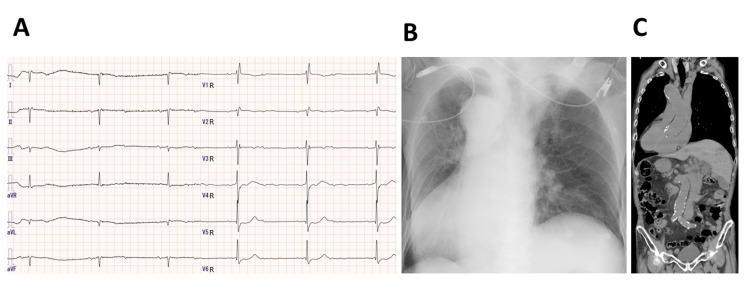
Examinations on admission A: The 12-lead ECG of the patient on admission. The precordial leads were placed on V1R to V6R. B: Chest X-ray of the patient on admission (AP supine view). C: Coronal CT scan of the chest and abdomen showing dextrocardia and situs inversus. ECG, electrocardiogram; CT, computed tomography; AP, anteroposterior.

A 94-year-old male was transferred to our hospital due to malaise, loss of appetite, and bradycardia with a heart rate of 30 beats per minute. He experienced syncope a few months prior to presentation. He had been treated with an angiotensin II receptor blocker for hypertension by his family doctor. At the age of 70 years he underwent a gastrectomy for gastric cancer. He received an operation for lumbar spinal stenosis at an age of 90. An electrocardiogram (ECG) demonstrated 2:1 atrio-ventricular block with a ventricular rate of 34 bpm (Figure 1A). Chest radiography and computed tomography (CT) revealed the existence of dextrocardia with situs inversus (Figure 1B, 1C).

The patient received an emergency cardiac catheterization since the cardiac troponin T was positive. Coronary angiography showed no significant stenosis in the coronary arteries. A temporary pacemaker was inserted from the right internal jugular vein for treatment of symptomatic bradycardia. His symptoms and pulmonary congestion improved after VVI pacing at 70 bpm, and an elective implantation of the permanent pacemaker was scheduled. Echocardiography demonstrated a normal left ventricular function and no significant valve dysfunction or other cardiac anomalies. On the next day after admission, the patient removed the pacing lead of temporary pacemaker unintentionally due to delirium during the daytime. His daily cognitive ability was normal. We discussed the treatment option as to whether to insert a temporary pacemaker again and implant a transvenous pacemaker electively or to implant a leadless pacemaker directly. The medical team explained the family members about the possible repetition of removal of the temporally pacemaker before implantation of transvenous pacemaker. Although, in patients with atrio-ventricular block lacking atrial fibrillation, dual-chamber pacing, not single-chamber pacemaker, is recommended, his family requested to implant a leadless pacemaker.

**Figure 2:**
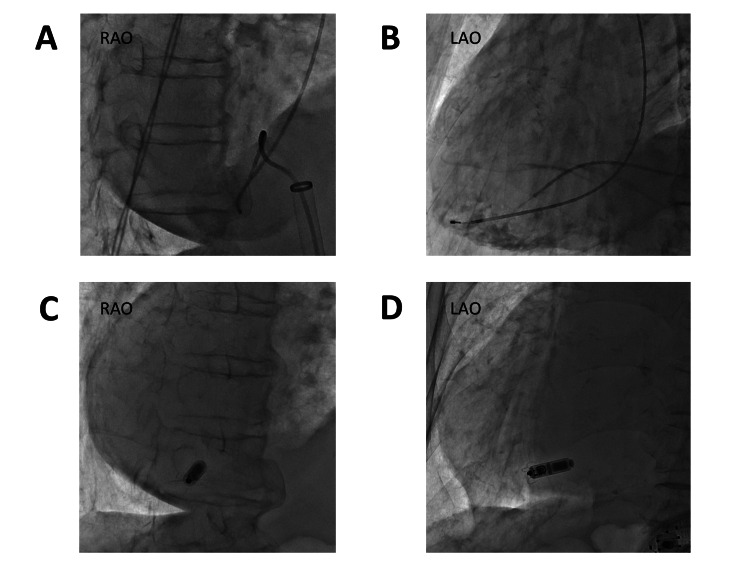
The implantation of the leadless pacemaker A: Right ventriculography in the RAO 30° view before the implantation of the leadless pacemaker. B: Right ventriculography in the LAO 50° view before the implantation of the leadless pacemaker. C: Fluoroscopic images in the RAO 30° view during the implantation of the leadless pacemaker. D: Fluoroscopic images in the LAO 50° view during the implantation of the leadless pacemaker. LAO, left anterior oblique; RAO, right anterior oblique.

**Figure 3:**
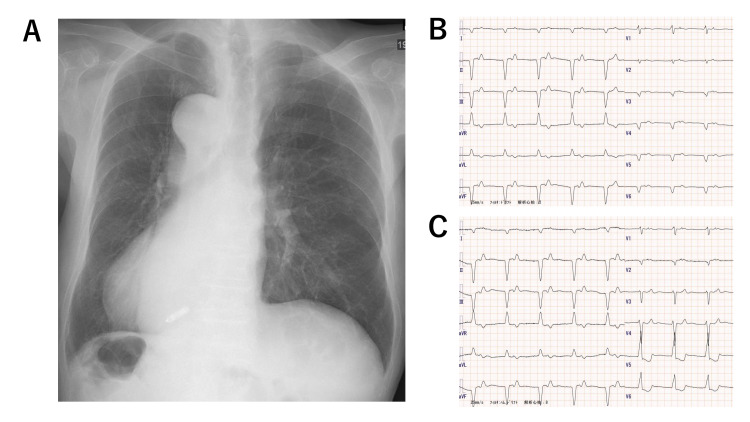
Examinations after the implantation A: Chest X-ray of the patient after implantation of a leadless pacemaker (PA standing view). B: The 12-lead ECG of the patient after the implantation of the leadless pacemaker. C: The 12-lead ECG of the patient after the implantation of the leadless pacemaker. Leads V3-6 were replaced with V3-6R. ECG, electrocardiogram; PA, posteroanterior.

The implantation of a leadless pacemaker was performed under fluoroscopy after the right ventriculography (Figure 2A, 2B). The procedure was performed according to the manufacturer’s standard recommendations. Due to the existence of dextrocardia and situs inversus, the procedure required additional time. To obtain a stable fixation and an adequate pacing threshold on the mid-septum of the right ventricle, we had to deploy and retract the device 6 times. The risk factors for the cardiac injury were advanced age, low body mass index, and absence of atrial fibrillation. Finally, we could successfully implant it on the septum close to the apex of the right ventricle, and obtain a good sensing parameter and pacing threshold (Figure 2C, 2D). In the pull-and-hold test, at least two tines were firmly fixed. The final parameters at the end of the procedure were an impedance of 570 ohms, R-wave sensing of 4.3 mV, and right ventricular pacing threshold of 1.0 V at 0.24 ms. The pacing threshold further improved to 0.38 V at 0.24 ms on the next day. The clinical course after the implantation was uneventful and the patient was discharged without any complications. The chest radiograph (Figure 3A) and ECG (Figure 3B) after the implantation are shown in Figure 3.

## Discussion

The leadless pacemaker (MicraTM, Medtronic, Minneapolis, MN) is a single-chamber ventricular pacemaker with a volume of 0.8 cm^3^, length of 25.9 mm, and weight of 2.0 g. Its functionality and features are similar to those of existing VVI(R) pacemakers with a longevity of 12.5 years [2,3]. The implantation procedure requires a steerable catheter delivery system, which is inserted into the right ventricle via a femoral vein with the use of a 23-French introducer. Previous studies have shown that Micra can be implanted feasibly and safely, and compared to traditional pacemakers, major complications have occurred 63% less frequently [4].

Dextrocardia with situs inversus is a rare condition that is characterized by abnormal mirror image positioning of the heart and other organs. Although majority of affected individuals are asymptomatic, some individuals may have congenital heart anomalies or ciliary dyskinesia referred to Kartagener syndrome. Device implantations sometimes are complicated by anatomical anomalies of the heart. During the leadless pacemaker implantation, implanting tools are not made for those conditions and the procedures using existing delivery systems are challenging.

So far, successful implantation of leadless pacemakers in patients with dextrocardia and situs inversus has been rarely reported. To the best of our knowledge, Regibus et al. reported one case of a leadless pacemaker implantation in a 36-year-old male patient with dextrocardia and situs inversus [5]. A transcatheter lead was extracted due to endocarditis. In addition, Cont et al. reported a case of a leadless pacemaker implantation in an acquired dextroposition after a right-sided pneumectomy [6]. Though rare, cardiac electrophysiologists may be also requested to perform intervention for cardiac arrhythmias in patients with this cardiac anomaly [7]. Implantation of cardiac devices or electrophysiological procedures must be performed with particular attention to avoid complications since all procedures are reversed due to mirror image in patient with dextrocardia and situs inversus.

## Conclusions

We implanted a leadless pacemaker for symptomatic high-degree atrio-ventricular block in an elderly patient with dextrocardia and situs inversus. Shorter time of bed-rest after the implantation and shorter hospital stay would be beneficial to elderly patients with a risk of delirium. However, a precise anatomical evaluation by fluoroscopy, venography, and right ventriculography would be important for grasping accurate anatomy and performing the procedure of implantation efficiently and safely.
